# Reporting of Accidents from Local Anesthetics

**Published:** 1918-03

**Authors:** Torald Sollmann, R. A. Hatcher

**Affiliations:** Chairman; Special Referee. 414 E. 26th St., New York City


### REPORTING OF ACCIDENTS FROM LOCAL ANESTHETICS

The Committee on Therapeutic Research of the Council on Pharmacy and Chemistry of the American Medical Association has undertaken a study of the accidents following the clinical use of local anesthetics, especially those following ordinary therapeutic doses. It is hoped that this study may lead to a better understanding of the cause of such accidents, and consequently to methods of avoiding them, or, at least, of treating them successfully when they occur.

It is becoming apparent that several of the local anesthetics, if not all of those in general use, are prone to cause death or symptoms of severe poisoning in a small percentage of those cases in which the dose used has been hitherto considered quite safe.

The infrequent occurrence of these accidents and their production by relatively small doses point to a peculiar hypersensitiveness on the part of those in whom the accidents occur. The data necessary for a study of these accidents are at present wholly insufficient, especially since the symptoms described in most of the cases are quite different from those commonly observed in animals even after the administration of toxic, but not fatal, doses.

Such accidents are seldom reported in detail in the medical literature, partly because physicians and dentists fear that they may be held to blame should they report them, partly, perhaps, because they have failed to appreciate the importance of the matter from the standpoint of the protection of the public.

It is evident that a broader view should prevail, and that physicians should be informed regarding the conditions under which such accidents occur in order that they may be avoided. It is also evident that the best protection against such unjust accusations, and the best means of preventing such accidents consist in the publication of careful detailed records when they have occurred, with the attending circumstances. These should be reported in the medical or dental journals when possible; but when, for any reason, this seems undesirable, a confidential report may be filed with Dr. R. A. Hatcher, 414 East Twenty-sixth Street, New York City, who has been appointed by the committee to collect this information.

If desired, such reports will be considered strictly confidential so far as the name of the patient and that of the medical attendant are concerned, and such information will be used solely as a means of studying the problem of toxicity of this this class of agents, unless permission is given to use the name.

All available facts, both public and private, should be included in these reports, but the following data are especially to be desired in those cases in which more detailed reports can not be made:

The age, sex and general history of the patient should be given in as great detail as possible. The state of the nervous system appears to be of especial importance. The dosage employed should be stated as accurately as possible; also the concentration of the solution employed, the site of the injection (whether intramuscular, perineurial or strictly subcutaneous), and whether applied to the mouth, nose or other part of the body. The possibility of an injection having been made into a small vein during intramuscular injection or into the gums should be considered. In such cases the action begins almost at once, that is, within a few seconds.

The previous condition of the heart and respiration should be reported if possible; and, of course, the effects of the drug on the heart and respiration, as well as the duration of the symptoms, should be recorded. If antidotes are employed, their nature and dosage should be stated, together with the character and time of appearance of the effects induced by the antidotes. It is important to state whether antidotes were administered orally or by subcutaneous, intramuscular or intravenous injection, and the concentration in which such antidotes were used.

While such detailed information, together with any other available data, are desirable, it is not to be understood that the inability to supply such details should prevent the publication of reports of poisoning, however meager the data, so long as accuracy is observed.

The committee urges on all anesthetists, surgeons, physicians and dentists the making of such reports as a public duty; it asks that they read this appeal with especial attention of the character of observations desired.

Therapeutic Research Committee of the Council on Pharmacy and Chemistry of the American Medical Association.

TORALD SOLLMANN, Chairman,
R. A. HATCHER, Special Referee.
414 E. 26th St., New York City.

